# Tanning a Chancre

**Published:** 1844-08-24

**Authors:** 


### TANNING A CHANCRE.

M. Ricord is unquestionably a man of crotchets. His vagaries are so manifold that he is a sort of "Pacha of many tales." His last is to tan a chancre, and to make it drunk—why, Heaven only knows, unless it is for the sake of making blockheads gape, and other people stare, a motive perfectly à la mode and French. But, for the process.

M. Ricord's favorite application is lint impregnated with aromatic wine. This is a decoction of sage, rosemary, thyme, hyssop, mint, &c. in eight times their weight of Burgundy wine. It destroys the contagious quality of the secretion from a chancre and *tans* the neighboring parts, rendering them less susceptible. M. Ricord has now and then found benefit from the addition of from one to six per cent. of tannin to the foregoing, to promote the latter purpose.—*Prov. Journ.*

What stuff is this! Tanning the skin around a chancre to prevent the spreading of infection! If it is received into the *system*, it is by the absorbents passing to the sore itself, and tanning the skin around cannot affect *them*. The spreading of the *mere sore* is prevented in one of two ways, or in both—by stimulating the part and exciting healthy action, or by acting on the system, and through it obtaining the same result. M. Ricord's wine and thyme, &c. are just a local stimulant and nothing else, and, no doubt, there are plenty as good or better—whilst the tanning is all a matter of moonshine. Et voilà tout.—*Med. Chir. Rev.*

